# Long-Term Safety Evaluation of Fluorescent Gold Nanoclusters Conjugated with α-Lipoic Acid: Insights from a Six-Month In Vivo Study

**DOI:** 10.3390/jfb16030089

**Published:** 2025-03-05

**Authors:** Yu-Wei Lai, Yi-Nan Lee, Hung-I Yeh, Yih-Jer Wu, Wen-Hsiung Chan, Shih-Wei Wang, Chao-Feng Lin, Chun-Hsuan Lin, Yun-Fang Chen, Ching-Hu Chung

**Affiliations:** 1 Center of General Education, University of Taipei, Taipei 100, Taiwan; dai77@tpech.gov.tw; 2 Division of Urology, Taipei City Hospital Renai Branch, Taipei 106, Taiwan; 3 Cardiovascular Center, Department of Internal Medicine, MacKay Memorial Hospital, Taipei 104, Taiwan; yinanlee168@gmail.com (Y.-N.L.); hiyeh@mmh.org.tw (H.-I.Y.); jacobyjwu@mmc.edu.tw (Y.-J.W.); p00899@mmc.edu.tw (C.-F.L.); 4 Department of Medicine, MacKay Medical College, New Taipei City 252, Taiwan; shihwei@mmc.edu.tw (S.-W.W.); p01781-632@mmc.edu.tw (C.-H.L.); 5 Department of Bioscience Technology and Center for Nanotechnology, Chung Yuan Christian University, Zhongbei Road, Zhongli District, Taoyuan City 320, Taiwan; whchan@cycu.edu.tw; 6 Institute of Biomedical Sciences, MacKay Medical College, New Taipei City 252, Taiwan

**Keywords:** chronic toxicity, fluorescent gold nanoclusters, α-lipoic acid

## Abstract

Background: Fluorescent gold nanoclusters conjugated with α-lipoic acid (FANCs) have shown great promise for drug development. In a previous study, FANCs did not show any acute or subacute toxicity under 0.6–20 μM/100 μL/25 g body weight in male and female ICR mice. However, the chronic toxicity of FANCs has not been studied. Aim of study: This study used oral administration of FANCs to determine the long-term safety profile and adverse effects in ICR mice. Methods: In vivo chronic toxicity was examined via oral administration of FANCs to male and female ICR mice. The daily food consumption, body weight, hematological profile, serum biochemical profile, organ coefficient, histopathological changes, and survival rate of the mice were calculated. Results: FANCs did not result in mortality due to chronic toxicity in both male and female mice. The animal behavior, body weight, hematological profile, serum biochemical profile, and organ coefficient showed no treatment-related malignant changes. This indicates that FANCs do not cause liver, renal, or other organ damage. Conclusions: These results indicate that the no-observed-adverse-effect level (NOAEL) is 20 μM/100 μL/25 g for 6 months of treatment in male and female ICR mice.

## 1. Introduction

Nanotechnology involves the study of matter at the atomic and molecular levels, focusing on structures ranging from 1 to 100 nm in size [1]. The size of nanoparticles will affect biokinetics, as smaller nanoparticles can increase the time of circulation in vivo [2]. Ultrasmall Au nanoparticles with a core size of less than 2 nm are called Au nanoclusters, consisting of less than 100 gold atoms, and are more stable and controllable than larger nanoparticles [3]. Surface engineering represents a forefront research topic in the field of metal nanoparticles, as the ligand layer on the nanoparticles’ surface is a crucial and determinant component for fundamental and applied research [4,5]. The effects of surface ligands become even more prominent in ultrasmall metal nanoparticles [6]. In addition to offering effective protection for metal nanoclusters (NCs) in solution, the design of surface ligands is crucial for determining their physical and chemical properties.

The surface ligands of FANCs are composed of α-lipoic acid (LA), which can be synthesized and used as a cofactor in mitochondrial enzymes and other multienzyme complexes [7,8,9]. LA has excellent antioxidant capacity, and its content in the body gradually decreases with age. Many studies have shown that LA has beneficial effects in preventing and treating many aging-related diseases (e.g., neurodegenerative diseases, metabolic disorders, atherosclerosis, cancer, kidney disease, infertility, and skin aging) [10]. In previous research, LA was reduced to dihydrolipoic acid (DHLA) for capping ligands [11]. A total of 1 M of FANCs contains 200–250 M of Au and 37–80 M of DHLA [12]. LA can enhance cholesterol efflux, suppress H_2_O_2_-induced pro-inflammatory factors (ICAM-1 and VCAM-1), and decrease the risk of aortic atherosclerosis [12,13,14]. In a comparison of the effects of molar concentration, FANCs potentiated LA’s anti-inflammatory and anti-atherosclerotic effects by 100–1000-fold [12].

Gold has traditionally been considered inert and biocompatible; due to its physicochemical properties and high surface area, gold nanoparticles are increasingly used in biomedical research [15]. Gold nanoclusters (AuNCs) can be synthesized using various ligands, including sulfhydryl small molecules, dendritic macromolecules, and proteins, among others [16]. Ligands not only provide protection for AuNCs but also enable direct interaction with target molecules [17]. Therefore, the properties of ligands significantly influence the effects of AuNCs. Ligand-protected AuNCs generally exhibit good biocompatibility and have diversified applications [18]. AuNCs possess tumor penetration and photoresponsive abilities, making them suitable for cancer photodynamic therapy and photothermal therapy [19]. Their high stability, stable fluorescence, and anti-bleaching properties also make AuNCs valuable tools for detection and imaging [16]. Additionally, AuNCs (≤2 nm) are smaller than gold nanoparticles and have demonstrated antimicrobial potential [20]. However, this also raises concerns about their potential to cause cellular damage, leading to cytotoxicity, genotoxicity, and inflammatory responses. Studies have demonstrated that smaller AuNPs can more readily penetrate cells and accumulate in various organs, such as the liver, spleen, and brain, thereby posing potential health risks [21,22].

In previous studies, our team developed a one-pot synthetic strategy to produce water-soluble fluorescent gold nanoclusters (FANCs), which show great promise as imaging agents for biomedical and cellular applications [11,23]. The ligand used in these FANCs is DHLA. In addition to cell labeling, FANCs have been shown to attenuate human aortic endothelial cell senescence and preserve their proliferation [24]. Furthermore, FANCs reduce IL-1 and TNF-α levels induced by LPS in mouse serum, demonstrating anti-inflammatory effects [24]. Another study found that DHLA-AuNCs can regulate neuronal inflammation and promote neuronal regeneration [25]. FANCs have also demonstrated anti-atherosclerotic effects by inhibiting intestinal cholesterol absorption and reducing macrophage attachment to endothelial cells [12]. However, inorganic nanoparticles are difficult to biodegrade and may induce long-term toxicity, which hinders their clinical application [26]. To evaluate the safety of FANCs, acute and subacute toxicity tests were conducted in our previous study.

The acute and subacute toxicity results revealed that FANCs exhibited no toxic effects over 14 days of administration [27]. However, drugs or health foods used to prevent atherosclerosis usually require long-term use. Therefore, the chronic toxicity of FANCs is critical for its development as a therapeutic agent for chronic diseases. This study aimed to evaluate the chronic toxicity of FANCs through oral administration in a mouse model.

## 2. Results

### 2.1. Behavior, Body Weight, and Food Intake

**Figure 1:**
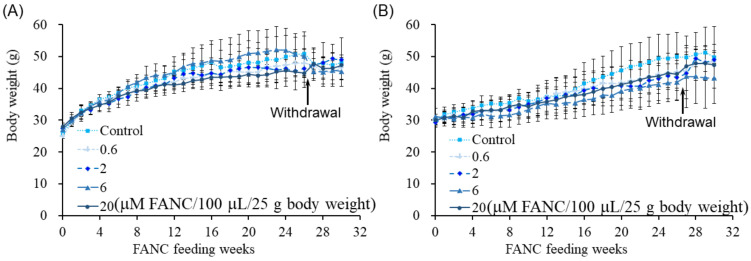
Body weight of ICR mice after daily feeding with FANCs for 6 months, followed by 4-week withdrawal. Body weight of (**A**) male and (**B**) female mice. Data are expressed as mean ± SD (*n* = 8; withdrawal: *n* = 3).

**Figure 2:**
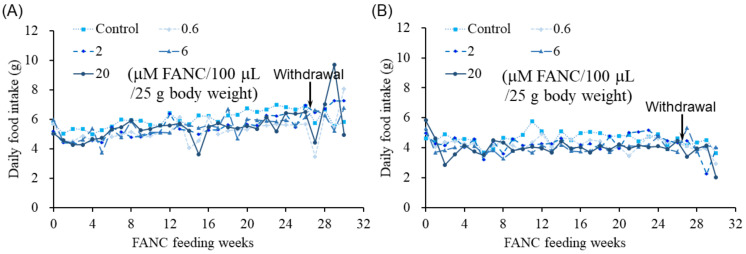
Daily food intake of ICR mice after daily feeding with FANCs for 6 months. Daily food intake of (**A**) male and (**B**) female mice. Food intake was calculated according to each mouse’s daily consumption. Data are expressed as mean ± SD.

To assess the potential toxicity of FANCs, male and female ICR mice were administered FANCs daily via oral gavage for six months, followed by a four-week withdrawal period to study their toxic reaction recovery. Doses of 0.6, 2, 6, and 20 μM of FANCs per 100 μL per 25 g of body weight were used. Throughout the experimental period, FANC administration had no observable effects on behavior or survival rates, and all mice appeared healthy. The body weight of the male and female mice increased with age across all groups, with no significant differences observed between the control and FANC-treated groups (Figure 1). Although daily food intake fluctuated, no long-term differences were detected between the FANC-treated groups and the control group (Figure 2).

### 2.2. Hematological Parameters

Hematological parameters, including RBC, HGB, HCT, MCV, MCH, MCHC, RDW, WBC, LYM%, LYM#, and PLT, showed no significant changes in the ICR mice after daily FANC administration for six months compared with the control group (Table 1). During the six-month feeding period, a small amount of blood was collected every four weeks for hematological parameter monitoring. No significant differences were observed between the FANC-treated and control groups throughout the study (Tables S1–S7). Furthermore, FANC administration for six months, followed by a four-week withdrawal period, did not affect hematological parameters (Table S8).

### 2.3. Biochemical Parameters

To evaluate liver and kidney function, biochemical parameters, including GOT, GPT, BUN, and CRE, were measured in the ICR mice after daily FANC administration for six months; the results are shown in Table 2. To assess potential tissue and organ damage, serum LDH levels were also analyzed. The results indicated that only the 20 μM FANC/100 μL/25 g body weight dose in female mice significantly lowered GOT and LDH levels compared with the control group.

### 2.4. Organ Weight

**Figure 3:**
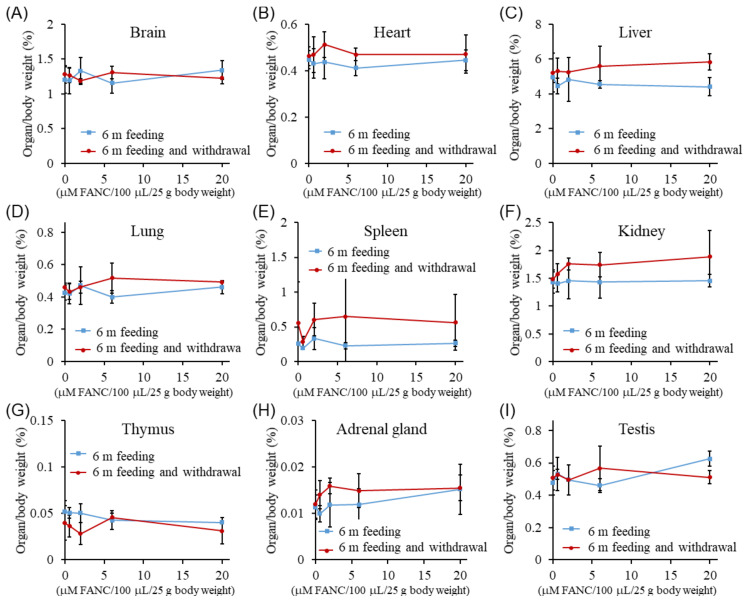

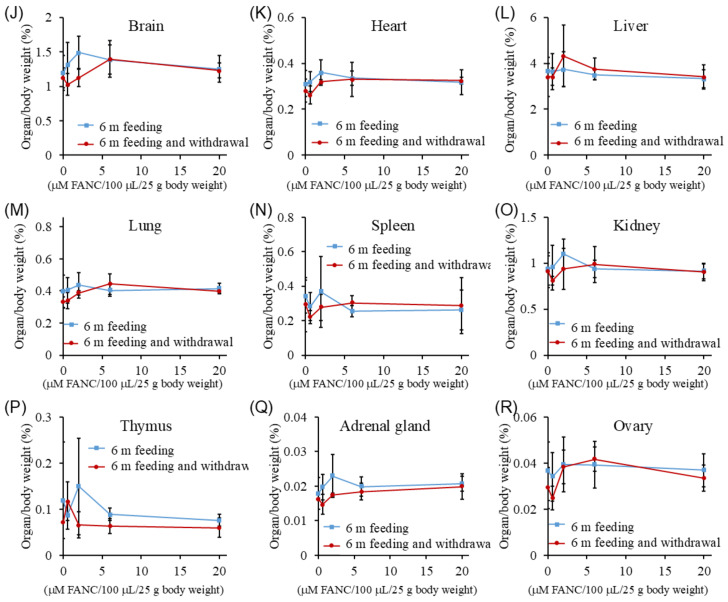
Changes in organ weight (%) of ICR mice after daily feeding with FANCs for 6 months, followed by 4-week withdrawal. Organ weight (%) of (**A**–**I**) male and (**J**–**R**) female mice. Organ weight% = (organ weight/body weight) × 100. Data are expressed as mean ± SD (6 m feeding: *n* = 5; withdrawal: *n* = 3).

The organ weights (%) of the ICR mice after daily FANC administration for six months, followed by a four-week withdrawal period, are presented in Figure 3. The organ weights of the brain, heart, liver, lungs, spleen, kidneys, thymus, adrenal glands, testes, and ovaries were similar between male and female mice across all experimental groups, with no significant differences compared with their respective control group. These findings suggest that FANC administration at doses below 20 μM FANC/100 μL/25 g body weight did not induce noticeable changes in organ weights.

### 2.5. Histopathological Changes

**Figure 4:**
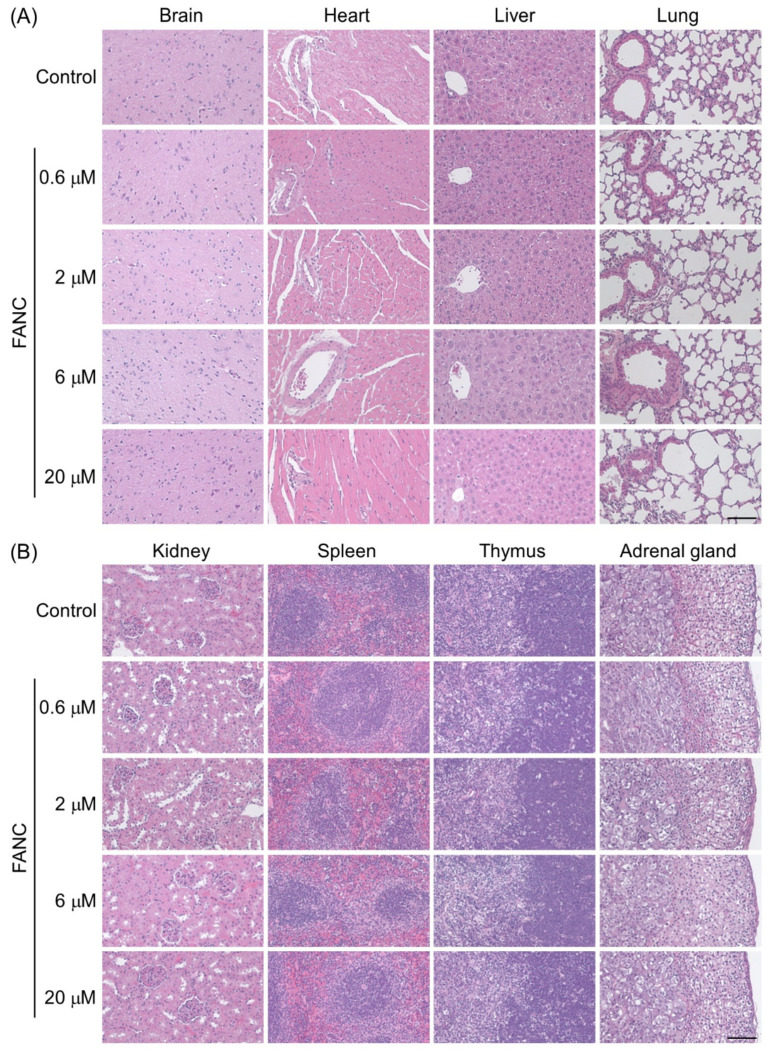

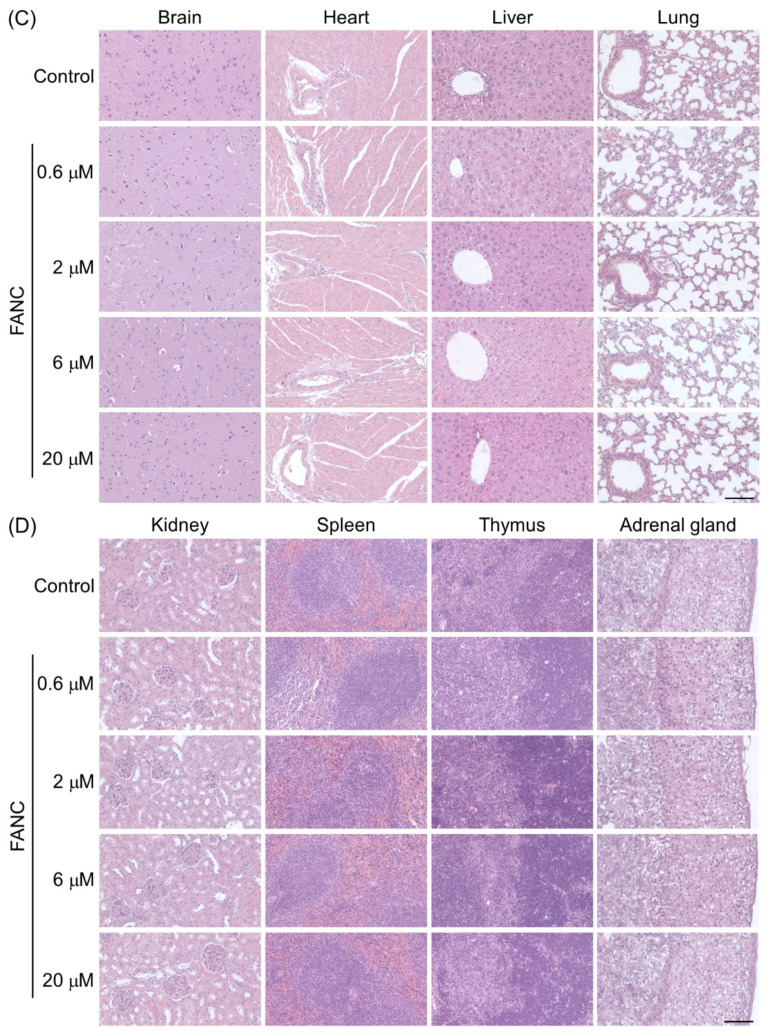

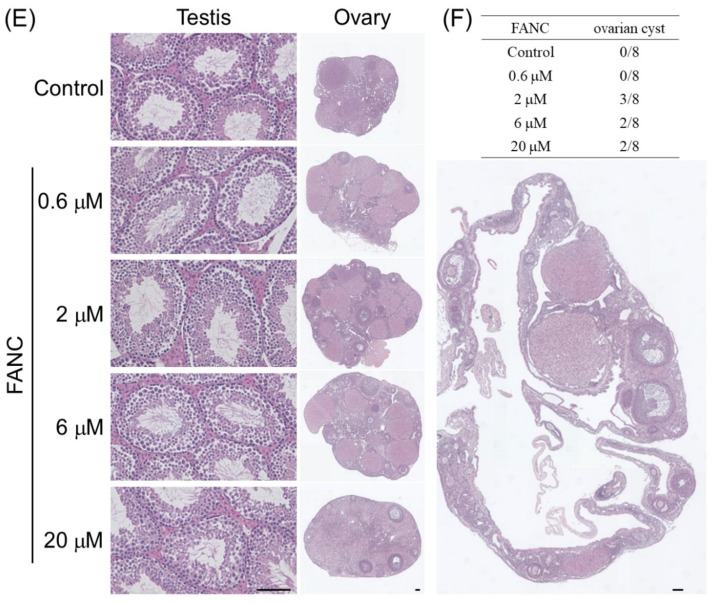
Representative H&E-stained histological sections of tissues from ICR mice after feeding with FANCs for 6 months. Sections of brain, heart, liver, lung, kidney, spleen, thymus, and adrenal gland from (**A**,**B**) male and (**C**,**D**) female mice. (**E**) Sections of testis from male and sections of ovary from female mice. (**F**) Incidence and representative image of an ovarian cyst from an FANC-treated group. Scale bar = 100 μm.

The histopathological changes in the brain, heart, liver, lung, kidney, spleen, thymus, adrenal gland, testis, and ovary sections stained with hematoxylin–eosin are presented in Figure 4. No treatment-related histopathological changes were observed in the brain, heart, liver, lung, kidney, spleen, thymus, adrenal gland, or testis. FANC administration for six months did not induce inflammation or fibrosis in the brain, heart, liver, lungs, or kidneys. Glomerular atrophy was not detected in any group. The spleen in all groups exhibited normal white (deeper purple areas) and red pulp areas. The thymus displayed normal medulla and cortex structures (deeper purple areas) across all groups. The histological structures of the testes were comparable to those of the control group, with germ cells and normal seminiferous tubules. The ovaries in both the control and FANC-treated mice displayed normal ovarian tissue and follicles at various developmental stages, including primary, secondary, and Graafian follicles; corpus luteum; and remnants of the corpus albicans (Figure 4E). However, it should be noted that some female mice in the groups administered FANCs at doses of 2, 6, and 20 μM FANC/100 μL/25 g body weight developed unilateral ovarian cysts (Figure 4F). The typical ovary size in this study was approximately 2–3 mm, whereas the ovarian cysts measured 5–10 mm. The ovarian cysts observed were smooth, thin-walled, and mostly translucent, containing clear fluid. Only two mice had cyst fluid with blood: one in the 2 μM FANC group and one in the 6 μM FANC group. Representative sections of the ovarian cysts stained with hematoxylin–eosin are shown in Figure 4F. The cysts were found to compress the ovarian tissue into a thin wall, but follicles at various developmental stages—including primary, secondary, and Graafian follicles, as well as corpus luteum and remnants of the corpus albicans—were still present. These findings suggest that the ovarian cysts are likely functional, simple follicular cysts.

## 3. Discussion

In previous acute and subacute toxicity studies, FANCs did not cause any morbidity or mortality in male or female ICR mice. Evaluating the potential of FANCs for long-term use and determining a safe dose require an examination of their toxicological profile. In this study, FANCs were administered to male and female ICR mice in a six-month oral gavage experiment, followed by a four-week withdrawal period. Doses of 0.6, 2, 6, and 20 μM FANC/100 μL/25 g body weight were tested. Behavioral observations, survival rates, and body weight measurements revealed no significant differences between the FANC-treated and control groups. Daily food intake and hematological parameters, including RBC, HGB, and WBC, remained unaffected throughout the study. Biochemical analysis of liver and kidney function showed no significant alterations, except for reduced GOT and LDH levels in female mice treated with the highest dose (20 μM FANC). Organ weight comparisons and histopathological examinations of major organs, including the brain, heart, liver, kidneys, and spleen, indicated no treatment-related changes, inflammation, or fibrosis. However, some female mice in the 2, 6, and 20 μM FANC groups developed unilateral ovarian cysts that were smooth and thin-walled, containing clear or blood-tinged fluid. Although the cysts compressed the ovarian tissue, normal follicular structures were maintained. These findings suggest that FANC administration up to 20 μM FANC/100 μL/25 g body weight induces minimal toxicity, but further investigation is warranted regarding ovarian cyst formation in female mice.

Previous studies have indicated that females are more sensitive than males to toxicants and drugs [28,29]. This gender variation is primarily attributed to hormonal regulation, which can influence the activity of various proteins or enzymes [30]. In this study, no toxic reactions were observed in male mice. However, in the FANC-treated female groups, the incidence of ovarian cysts was higher compared with the control group. This finding suggests that long-term use of FANCs may affect hormonal regulation in females, potentially leading to the development of ovarian cysts. Ovarian cysts are fluid-filled sacs that form on or within the ovaries, often arising due to hormonal imbalances, pathological conditions, or external factors. Ovarian cysts are potentially influenced by gonadotropins [31]. Occasionally, blood from the vascular theca zone may leak into the cyst, forming follicular hematomas [31]. For example, the fertility drug clomiphene is associated with a higher incidence of ovarian cysts because this drug may trigger the development of multiple follicles in the ovaries [32]. Ovarian cysts are generally harmless and functional in nature, often degenerating spontaneously within a few months [33,34]. As there were no significant differences in behavioral observations, daily food intake, or hematological parameters in the female mice treated with the highest dose (20 μM FANC) compared with the control mice, these results suggest that the FANC-induced ovarian cysts may be benign. In our previous study, no ovarian abnormalities were identified in acute and subacute toxicity tests [27]. Based on these observations, we conclude that the formation of ovarian cysts is not a toxic reaction, although their occurrence may be somewhat related to the FANC treatment concentration.

Another potential toxic reaction associated with FANC administration was the observed reduction in GOT and LDH levels at the highest dose in female mice. While elevated serum GOT and LDH levels are commonly linked to tissue damage and various pathological conditions, decreased levels are less frequently encountered and are often deemed clinically insignificant. However, in specific contexts, such as unexplained muscle weakness or exercise intolerance, the possibility of genetic deficiencies in LDH should be investigated [35]. Additionally, conditions characterized by reduced metabolic activity, including malnutrition or chronic diseases, may also contribute to lower GOT and LDH levels. Given that no significant differences were observed in genetic factors, behavioral patterns, daily food intake, or hematological parameters between the female mice treated with the highest dose and the control mice, these findings suggest that the reduced GOT and LDH levels are likely clinically insignificant.

Our findings suggest that FANCs exhibit a promising safety profile for chronic exposure. However, future studies should thoroughly investigate their pharmacokinetics, long-term biodistribution, and accumulation to fully explore their potential in clinical applications. The limitations of this study were that the synthesized FANC concentration was almost 20 μM, and we were unable to study the toxicity of FANCs at higher concentrations. In addition, this concentration was 3.3 times higher than the anti-atherosclerosis dose in the previous study [12].

## 4. Conclusions

The results indicate no significant adverse effects of FANCs on general health, survival rates, or organ function, with a no-observed-adverse-effect level (NOAEL) determined at 20 μM/100 μL/25 g body weight for both sexes. However, some female mice developed functional ovarian cysts at higher doses, highlighting potential sex-specific responses. Biochemical analysis revealed minor changes in GOT and LDH levels in females treated with the highest dose, though these were deemed clinically insignificant. Histopathological examination showed no evidence of inflammation or fibrosis in vital organs. These findings demonstrate FANCs’ minimal toxicity under chronic exposure, supporting their potential for long-term therapeutic use. Further investigations into their biodistribution, pharmacokinetics, and sex-specific effects are recommended to ensure comprehensive safety profiling.

## 5. Materials and Methods

### 5.1. Synthesis of FANCs via a One-Pot Synthetic Strategy

The FANCs used in this study were produced by GOLDRED NANOBIOTECH CO., LTD. (Taoyuan City, Taiwan). FANCs are gold nanoclusters with a diameter of 1.56 ± 0.3 nm, featuring a negatively charged surface modification; they were synthesized via a one-pot method as previously described [11,23]. Briefly, gold nanoparticles (6 nm; stabilized with didodecyldimethylammonium bromide) were treated with AuCl₃ to produce nanoclusters, followed by ligand exchange with DHLA. Excess reagents were removed through a series of precipitation, ultracentrifugation, reconstitution, and centrifuge filtration (30 kDa molecular weight cutoff), with the buffer replaced by deionized water. Finally, thermal treatment for 24 h increased the quantum yield of FANCs to approximately 7%. The resulting FANC solution was a colloidally stable, dark-brown transparent liquid showing red fluorescence under UV light excitation.

### 5.2. Animals and Ethical Statements

The Institute of Cancer Research (ICR) mice used in this study were obtained from BioLASCO Taiwan. All animal experiments were approved by the Laboratory Animal Use Committee of MacKay Medicine College (approval number: A1080016) and conducted in accordance with the Guidelines for Care and Use of Experimental Animals (Canadian Council on Animal Care, Ottawa, 1984). The animals were kept in the MacKay Medicine College laboratory animal room. The mice were provided with an MFG laboratory animal diet (Oriental Yeast Co., Ltd., Chiba, Japan) and water ad libitum. They were housed in standard polypropylene cages with wire-grid tops, aspen shavings (NEPCO), beta chips (NEPCO), and a tube (environmental enrichment) and maintained under a 12 h light/12 h dark cycle. Before the study, all animals were acclimatized for a week to minimize stress before the study began.

### 5.3. Chronic Toxicity Test

**Figure 5:**
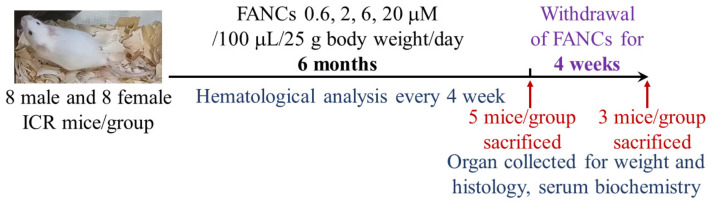
The schematic diagram of the study design.

The chronic toxicity test followed the guidelines of the International Council for Harmonisation of Technical Requirements for Pharmaceuticals for Human Use (ICH). Forty male and forty female 7-week-old mice were randomly divided into five groups (*n* = 8): control (water), 0.6, 2, 6, and 20 μM FANC/100 μL/25 g body weight (equivalent to 0.16, 0.53, 1.57, and 5.26 μg FANC/kg). FANCs were administered orally via gavage daily for six months. Physiological and behavioral changes, toxicological signs, and mortality were observed daily, and food intake and body weight were measured weekly. Blood samples were collected every four weeks for hematological analysis. After six months, five mice per group were randomly selected and sacrificed for organ weight measurement, serum biochemistry, and histopathological analysis. Organ tissues were fixed in 10% neutral-buffered formaldehyde, embedded in paraffin, sectioned at 2 μm, and stained with hematoxylin–eosin for histopathological evaluation. The remaining three mice in each group underwent a four-week withdrawal period, after which they were sacrificed for toxic reaction recovery assessment (Figure 5).

### 5.4. Hematological Analysis

The mice were anesthetized with isoflurane, followed by cardiac puncture exsanguination to collect blood samples. The collected blood was immediately mixed with 3.2% sodium citrate in a ratio of 9:1. The blood samples were analyzed using a hematological analyzer (Sysmex KX-21 Hematology Analyzer; Mundelein, IL, USA) to measure red blood cells (RBCs), hemoglobin (HGB), hematocrit (HCT), mean corpuscular volume (MCV), mean corpuscular hemoglobin (MCH), mean corpuscular hemoglobin concentration (MCHC), red blood cell distribution width (RDW), white blood cells (WBCs), lymphocyte percentage (LYM%), lymphocyte count (LYM#), and platelets (PLTs).

### 5.5. Serum Biochemical Analysis

The collected blood was allowed to stand at room temperature for 30 min and then centrifuged (3000 rpm for 10 min at 4 °C) to collect serum. The serum samples were analyzed using a Fuji Dri-Chem 4000i biochemical analyzer to measure creatinine (CRE), blood urea nitrogen (BUN), glutamic–pyruvic transaminase (GPT), glutamic–oxaloacetic transaminase (GOT), and lactate dehydrogenase (LDH). This analyzer utilizes specially prepared reagent strips, with a small volume of serum applied to the reagent strip for reactions, and the analyzer measures the color change or light absorption resulting from the chemical reactions. The CRE assay was based on the Jaffe method, which works by measuring creatinine’s reaction with alkaline picrate. The BUN assay was based on the urease method, wherein urease catalyzes the conversion of urea to ammonia. The level of ammonia was then detected using a colorimetric method to measure the change in absorbance. GPT catalyzes the conversion of alanine and α-ketoglutarate to pyruvate and glutamate. This conversion process also results in the reduction of NAD^+^ to NADH, whose change in absorbance can be measured. GOT catalyzes the transfer of an amino group from aspartate to α-ketoglutarate. This process involves the oxidation of NADH, with a decrease in absorbance. LDH activity was monitored by examining the conversion of lactate to pyruvate, as well as the reduction of NAD^+^ to NADH.

### 5.6. Statistics

Continuous data are expressed as mean ± standard deviation (SD) by Excel. Comparisons between two groups were performed using a two-tailed *t*-test, while one-way analysis of variance (ANOVA), followed by Newman–Keuls post hoc tests, were used for multiple comparisons. All *p*-values were two-sided, with *p* < 0.01 considered statistically significant.

## Figures and Tables

**Table 1:** Changes in hematological parameters of ICR mice fed with FANCs for 6 months.

	μM FANC/100 μL/25 g Body Weight				
**Male**	**0**	**0.6**	**2**	**6**	**20**
RBC	6.1 ± 0.8	6.4 ± 0.5	6.9 ± 0.2	6.2 ± 0.4	6.3 ± 0.4
HGB	11 ± 1	11 ± 1	12 ± 1	12 ± 1	11 ± 1
HCT	35 ± 5	36 ± 2	38 ± 2	34 ± 1	35 ± 3
MCV	57 ± 1	56 ± 1	55 ± 3	55 ± 2	56 ± 2
MCH	18 ± 2	18 ± 1	17 ± 2	19 ± 1	18 ± 2
MCHC	32 ± 4	32 ± 2	31 ± 2	34 ± 1	31 ± 3
RDW	14 ± 2	12 ± 1	15 ± 5	16 ± 1	15 ± 1
WBC	5.4 ± 1.2	5.8 ± 3.9	4.1 ± 2.6	5.7 ± 4.5	4.8 ± 1.4
LYM%	73 ± 11	80 ± 4	63 ± 17	61 ± 16	78 ± 23
LYM#	3.8 ± 0.7	4.6 ± 3.1	2.7 ± 1.8	3.2 ± 1.8	3.1 ± 1.5
PLT	835 ± 198	817 ± 374	729 ± 115	760 ± 168	800 ± 152
**Female**	**0**	**0.6**	**2**	**6**	**20**
RBC	6.6 ± 0.7	6.8 ± 0.6	6.7 ± 0.5	6.9 ± 0.2	7.1 ± 0.3
HGB	11 ± 1	11 ± 1	11 ± 1	11 ± 1	11 ± 1
HCT	36 ± 4	35 ± 3	36 ± 3	36 ± 2	37 ± 2
MCV	54 ± 1	52 ± 1	54 ± 1	53 ± 3	52 ± 2
MCH	17 ± 1	16 ± 1	17 ± 0	16 ± 1	16 ± 1
MCHC	31 ± 1	30 ± 1	31 ± 0	30 ± 1	30 ± 1
RDW	29 ± 1	28 ± 1	26 ± 4	28 ± 1	28 ± 1
WBC	2.2 ± 1.2	2.4 ± 1.4	2.5 ± 0.8	3.1 ± 0.7	2.7 ± 0.8
LYM%	92 ± 9	77 ± 9	85 ± 12	82 ± 8	81 ± 14
LYM#	2.4 ± 1.0	3.7 ± 2.3	2.1 ± 0.5	2.5 ± 0.6	2.2 ± 0.7
PLT	1051 ± 300	1067 ± 141	975 ± 143	1002 ± 137	1065 ± 145

RBC: red blood cell (10^6^/μL); HGB: hemoglobin (g/dL); HCT: hematocrit (%); MCV: mean corpuscular volume (fL); MCH: mean corpuscular hemoglobin (pg); MCHC: mean corpuscular hemoglobin concentration (g/dL); RDW: red blood cell distribution width (%); WBC: white blood cell (10^3^/μL); LYM%: lymphocyte percentage (%); LYM#: lymphocyte count (10^3^/μL); PLT: platelet (10^3^/μL). Data are expressed as mean ± SD (*n* = 5).

**Table 2:** Changes in serum biochemistry with respect to liver and renal function of ICR mice fed with FANCs for 6 months.

**Male**	**GOT**	**GPT**	**LDH**	**BUN**	**CRE**
**(μM)**	**(U/L)**	**(U/L)**	**(U/L)**	**(mg/dL)**	**(mg/dL)**
0	49 ± 8	23 ± 9	263 ± 57	25 ± 6	0.29 ± 0.15
0.6	43 ± 7	26 ± 7	199 ± 64	23 ± 5	0.15 ± 0.08
2	54 ± 17	23 ± 7	222 ± 74	24 ± 2	0.23 ± 0.14
6	45 ± 6	19 ± 4	224 ± 29	25 ± 8	0.20 ± 0.10
20	60 ± 5	23 ± 5	268 ± 126	24 ± 5	0.17 ± 0.06
**Female**	**GOT**	**GPT**	**LDH**	**BUN**	**CRE**
**(μM)**	**(U/L)**	**(U/L)**	**(U/L)**	**(mg/dL)**	**(mg/dL)**
0	56 ± 11	22 ± 14	347 ± 29	24 ± 4	0.12 ± 0.03
0.6	60 ± 29	13 ± 6	447 ± 339	28 ± 4	0.19 ± 0.17
2	62 ± 25	11 ± 1	283 ± 112	25 ± 1	0.13 ± 0.04
6	60 ± 18	19 ± 11	369 ± 164	26 ± 4	0.17 ± 0.03
20	37 ± 5 *	10 ± 3	222 ± 57 *	22 ± 2	0.14 ± 0.05

GOT: aspartate aminotransferase; GPT: alanine aminotransferase; LDH: lactate dehydrogenase; BUN: blood urea nitrogen; CRE: creatinine. Data are expressed as mean ± SD (*n* = 5). * *p* < 0.01.

## Data Availability

The original contributions presented in this study are included in the article and Supplementary Material. Further inquiries can be directed to the corresponding author.

## Supplementary Materials

The following supporting information can be downloaded at https://www.mdpi.com/article/10.3390/jfb16030089/s1, Table S1: Hematological parameters of ICR mice before feeding with FANCs; Table S2: Changes in hematological parameters of ICR mice fed with FANCs for 4 weeks; Table S3: Changes in hematological parameters of ICR mice fed with FANCs for 8 weeks; Table S4: Changes in hematological parameters of ICR mice fed with FANCs for 12 weeks; Table S5: Changes in hematological parameters of ICR mice fed with FANCs for 16 weeks; Table S6: Changes in hematological parameters of ICR mice fed with FANCs for 20 weeks; Table S7: Changes in hematological parameters of ICR mice fed with FANCs for 24 weeks. Table S8: Changes in hematological parameters of ICR mice fed with FANCs for 6 months and a 4-week withdrawal period.

## Abbreviations

The following abbreviations are used in this manuscript:

FANCs	Fluorescent gold nanoclusters conjugated with α-lipoic acid
AuNCs	Gold nanoclusters
DHLA	Dihydrolipoic acid
ICR	Institute of Cancer Research
RBC	Red blood cell
HGB	Hemoglobin
HCT	Hematocrit
MCV	Mean corpuscular volume
MCH	Mean corpuscular hemoglobin
MCHC	Mean corpuscular hemoglobin concentration
RDW	Red blood cell distribution width
WBC	White blood cell
LYM%	Lymphocyte percentage
LYM#	Lymphocyte count
PLT	Platelet
CRE	Creatinine
BUN	Blood urea nitrogen
GPT	Glutamic–pyruvic transaminase
GOT	Glutamic–oxaloacetic transaminase
LDH	Lactate dehydrogenase
NOAEL	No-observed-adverse-effect level
