# Supplementary material for: Long-Term Safety Evaluation of Fluorescent Gold Nanoclusters Conjugated with α-Lipoic Acid: Insights from a Six-Month In Vivo Study
Source: J Funct Biomater. 2025 Mar 5;16(3):89. doi: 10.3390/jfb16030089 (PMC11942834; doi:10.3390/jfb16030089)
Supplement: Supplementary file 1 [file jfb-16-00089-s001.zip › jfb-3463611-supplementary.pdf]

Tables S1. Hematological parameters of ICR mice before feeding with FANC

	μM FANC/100 μL/25 g mice				
Male	0	0.6	2	6	20
RBC	6.8 ± 0.3	6.8 ± 0.4	6.6 ± 0.3	6.6 ± 0.2	6.8 ± 0.9
HGB	11.8 ± 0.7	12.3 ± 0.8	11.7 ± 0.7	11.6 ± 0.8	12.0 ± 0.4
HCT	39.4 ± 2.0	40.1 ± 2.9	38.6 ± 2.1	38.2 ± 1.8	40.3 ± 1.7
MCV	58.3 ± 1.6	59.0 ± 1.7	58.6 ± 1.6	58.2 ± 0.9	57.5 ± 1.0
MCH	19.4 ± 6.7	17.7 ± 0.4	17.8 ± 0.6	17.8 ± 1.0	18.2 ± 3.5
MCHC	33.4 ± 12.0	30.8 ± 2.8	30.4 ± 0.6	28.0 ± 7.6	31.7 ± 6.1
RDW	14.6 ± 0.9	14.5 ± 1.2	15.7 ± 1.0	14.3 ± 1.1	14.1 ± 0.5
WBC	6.6 ± 2.9	8.7 ± 2.9	8.7 ± 2.6	6.5 ± 3.1	6.9 ± 2.4
LYM%	67.9 ± 14.9	66.6 ± 9.5	60.4 ± 16.9	67.2 ± 9.5	54.5 ± 9.1
LYM#	4.4 ± 2.2	5.7 ± 1.9	5.7 ± 2.8	4.4 ± 2.2	2.9 ± 1.2
PLT	709 ± 99	659 ± 126	617 ± 118	768 ± 127	805 ± 109

	μM FANC/100 μL/25 g mice				
Female	0	0.6	2	6	20
RBC	7.5 ± 0.3	7.9 ± 0.8	7.8 ± 0.7	7.6 ± 0.3	7.9 ± 0.5
HGB	13.8 ± 0.6	14.3 ± 1.6	13.9 ± 1.0	13.9 ± 0.8	14.2 ± 0.8
HCT	43.8 ± 2.1	45.7 ± 4.9	45.4 ± 3.5	43.8 ± 1.8	44.4 ± 2.9
MCV	58.7 ± 1.1	58.1 ± 2.3	58.1 ± 1.9	57.7 ± 0.9	57.2 ± 1.1
MCH	18.5 ± 0.5	18.2 ± 1.0	17.9 ± 0.8	18.3 ± 0.9	18.0 ± 0.8
MCHC	31.4 ± 0.9	31.3 ± 0.8	30.8 ± 0.8	31.7 ± 1.4	31.9 ± 1.0
RDW	11.6 ± 0.4	11.9 ± 1.6	11.9 ± 1.7	11.7 ± 0.6	11.6 ± 0.5
WBC	8.5 ± 2.5	8.5 ± 2.5	8.0 ± 2.2	7.7 ± 2.3	7.3 ± 3.0
LYM%	76.8 ± 8.7	79.5 ± 8.1	75.4 ± 7.5	78.2 ± 8.2	73.9 ± 9.9
LYM#	6.5 ± 1.7	6.8 ± 2.2	6.0 ± 1.3	6.1 ± 2.1	5.6 ± 2.2
PLT	562 ± 198	552 ± 273	678 ± 124	676 ± 149	511 ± 177

RBC: red blood cell (10^6/μL); HGB: hemoglobin (g/dL); HCT: hematocrit (%); MCV: mean corpuscular volume (fL); MCH: mean corpuscular hemoglobin (pg); MCHC: mean corpuscular hemoglobin concentration (g/dL); RDW: red blood cell distribution width (%); WBC: white blood cell (10^3/μL); LYM%: lymphocyte percentage (%); LYM#: lymphocyte count (10^3/μL); PLT: platelets (10^3/μL). Data are expressed as the mean ± SD (n=8).

Tables S2. Changes of hematological parameters of ICR mice feeding with FANC for 4 weeks

μM FANC/100 μL/25 g mice

Male	0	0.6	2	6	20
RBC	7.5 ± 0.3	7.3 ± 0.3	7.2 ± 0.6	7.0 ± 0.8	7.4 ± 0.9
HGB	12.8 ± 0.6	12.1 ± 0.6	12.3 ± 1.0	12.1 ± 1.0	12.4 ± 1.6
HCT	39.7 ± 0.9	39.9 ± 1.5	38.6 ± 3.3	37.8 ± 5.2	38.5 ± 5.1
MCV	52.9 ± 1.1	54.4 ± 1.0	53.4 ± 1.6	54.2 ± 1.7	52.1 ± 1.3
MCH	17.1 ± 0.6	16.5 ± 0.5	17.1 ± 0.5	17.6 ± 2.6	16.7 ± 0.6
MCHC	32.3 ± 1.0	30.7 ± 0.7	32.0 ± 0.6	32.6 ± 5.6	32.1 ± 0.5
RDW	12.0 ± 5.0	11.5 ± 0.4	12.1 ± 0.5	11.6 ± 0.5	12.5 ± 1.2
WBC	5.2 ± 3.4	5.5 ± 3.3	2.4 ± 1.3	2.5 ± 1.7	4.8 ± 2.0
LYM%	63.2 ± 19.1	57.1 ± 8.7	70.3 ± 14.7	80.8 ± 10.1	67.5 ± 11.3
LYM#	2.5 ± 1.0	2.0 ± 0.7	1.4 ± 0.5	1.9 ± 1.2	2.6 ± 1.0
PLT	488 ± 158	561 ± 203	641 ± 256	650 ± 147	601 ± 207

RBC: red blood cell (10^6/μL); HGB: hemoglobin (g/dL); HCT: hematocrit (%); MCV: mean corpuscular volume (fL); MCH: mean corpuscular hemoglobin (pg); MCHC: mean corpuscular hemoglobin concentration (g/dL); RDW: red blood cell distribution width (%); WBC: white blood cell (10^3/μL); LYM%: lymphocyte percentage (%); LYM#: lymphocyte count (10^3/μL); PLT: platelets (10^3/μL). Data are expressed as the mean ± SD (n=8).

Tables S3. Changes of hematological parameters of ICR mice feeding with FANC for 8 weeks

	μM FANC/100 μL/25 g mice				
Male	0	0.6	2	6	20
RBC	7.9 ± 0.4	7.5 ± 0.4	7.6 ± 0.5	8.0 ± 0.3	7.5 ± 0.5
HGB	13.3 ± 0.4	13.0 ± 0.8	13.4 ± 0.8	13.7 ± 0.4	13.2 ± 0.8
HCT	44.6 ± 1.5	41.6 ± 2.1	42.1 ± 2.9	44.1 ± 1.3	41.2 ± 3.8
MCV	56.8 ± 1.6	55.3 ± 1.3	55.3 ± 1.1	55.1 ± 1.3	54.8 ± 1.5
MCH	17.0 ± 0.6	17.2 ± 0.4	17.6 ± 0.7	17.1 ± 0.4	17.6 ± 1.1
MCHC	30.1 ± 0.3	30.9 ± 0.8	31.9 ± 1.7	30.8 ± 0.5	31.5 ± 1.9
RDW	12.6 ± 0.5	12.7 ± 0.8	13.1 ± 1.9	12.0 ± 0.6	14.0 ± 2.6
WBC	7.6 ± 2.8	10.4 ± 2.8	7.7 ± 2.6	9.6 ± 1.8	-
LYM%	73.1 ± 8.8	62.9 ± 7.4	59.3 ± 5.7	66.4 ± 4.6	-
LYM#	5.7 ± 2.3	6.3 ± 1.6	4.3 ± 1.8	6.2 ± 1.1	-
PLT	936 ± 108	806 ± 140	711 ± 211	830 ± 200	869 ± 73

RBC: red blood cell (10^6/μL); HGB: hemoglobin (g/dL); HCT: hematocrit (%); MCV: mean corpuscular volume (fL); MCH: mean corpuscular hemoglobin (pg); MCHC: mean corpuscular hemoglobin concentration (g/dL); RDW: red blood cell distribution width (%); WBC: white blood cell (10^3/μL); LYM%: lymphocyte percentage (%);

LYM#: lymphocyte count ($10^3/\mu L$); PLT: platelets ($10^3/\mu L$). Data are expressed as the mean ± SD (n=8).

- machine malfunction, no data acquire.

Tables S4. Changes of hematological parameters of ICR mice feeding with FANC for 12 weeks

	µM FANC/100 µL/25 g mice				
Male	0	0.6	2	6	20
RBC	7.6 ± 0.5	7.8 ± 0.3	8.0 ± 0.4	7.9 ± 0.4	7.9 ± 0.3
HGB	13.3 ± 0.7	12.7 ± 1.3	14.0 ± 0.5	14.3 ± 1.0	14.0 ± 0.6
HCT	43.3 ± 3.1	43.7 ± 1.3	45.9 ± 1.9	44.4 ± 1.5	45.1 ± 2.2
MCV	57.0 ± 2.8	55.7 ± 1.4	57.1 ± 1.2	56.5 ± 1.2	57.0 ± 1.5
MCH	14.3 ± 9.0	16.2 ± 1.7	17.4 ± 0.7	18.2 ± 1.7	17.7 ± 0.7
MCHC	30.8 ± 0.9	29.2 ± 3.0	30.4 ± 0.8	32.2 ± 2.3	31.0 ± 1.0
RDW	13.5 ± 1.3	12.1 ± 0.8	11.8 ± 0.7	11.9 ± 0.5	13.5 ± 1.6
WBC	10.6 ± 5.4	8.0 ± 1.9	8.7 ± 2.8	8.8 ± 2.1	10.9 ± 2.6
LYM%	69.3 ± 11.6	57.7 ± 7.7	67.7 ± 13.6	66.1 ± 8.3	66.2 ± 12.7
LYM#	7.0 ± 3.5	4.7 ± 1.6	5.6 ± 1.6	5.8 ± 1.6	7.2 ± 2.2
PLT	638 ± 112	701 ± 170	665 ± 147	808 ± 105	827 ± 153

	µM FANC/100 µL/25 g mice				
Female	0	0.6	2	6	20
RBC	8.9 ± 0.2	8.6 ± 0.6	8.6 ± 0.3	8.6 ± 0.3	8.6 ± 0.4
HGB	15.0 ± 0.5	14.3 ± 0.5	14.4 ± 0.5	14.2 ± 0.7	14.2 ± 0.8
HCT	46.8 ± 1.1	45.0 ± 1.6	45.2 ± 1.0	45.7 ± 1.1	45.1 ± 2.6
MCV	54.0 ± 1.0	52.1 ± 2.4	52.9 ± 1.3	52.8 ± 1.8	52.5 ± 1.5
MCH	17.1 ± 0.4	16.7 ± 0.4	16.8 ± 0.8	16.7 ± 0.8	16.4 ± 0.9
MCHC	31.9 ± 1.0	30.9 ± 1.5	30.5 ± 3.2	31.6 ± 0.9	31.4 ± 0.9
RDW	28.6 ± 0.9	28.2 ± 0.6	27.1 ± 3.9	28.7 ± 0.6	28.4 ± 0.8
WBC	7.0 ± 3.7	7.9 ± 1.8	8.7 ± 3.3	7.6 ± 2.2	5.8 ± 1.8
LYM%	79.4 ± 13.2	69.6 ± 13.0	73.5 ± 14.2	70.5 ± 6.2	75.9 ± 8.8
LYM#	5.5 ± 3.0	5.4 ± 1.0	6.2 ± 2.3	5.3 ± 1.5	4.4 ± 1.5
PLT	659 ± 316	940 ± 152	755 ± 540	831 ± 372	920 ± 112

RBC: red blood cell ($10^6/\mu L$); HGB: hemoglobin (g/dL); HCT: hematocrit (%); MCV: mean corpuscular volume (fL); MCH: mean corpuscular hemoglobin (pg); MCHC: mean corpuscular hemoglobin concentration (g/dL); RDW: red blood cell distribution width (%); WBC: white blood cell ($10^3/\mu L$); LYM%: lymphocyte percentage (%); LYM#: lymphocyte count ($10^3/\mu L$); PLT: platelets ($10^3/\mu L$). Data are expressed as the

mean ± SD (n=8).

Tables S5. Changes of hematological parameters of ICR mice feeding with FANC for 16 weeks

	μM FANC/100 μL/25 g mice				
Male	0	0.6	2	6	20
RBC	7.3 ± 0.9	7.7 ± 0.6	7.7 ± 0.4	7.8 ± 0.4	7.8 ± 0.5
HGB	13.0 ± 1.2	13.7 ± 0.9	14.1 ± 0.9	13.9 ± 0.3	13.9 ± 0.8
HCT	42.7 ± 3.5	44.2 ± 3.8	44.1 ± 2.6	45.0 ± 1.8	45.7 ± 2.8
MCV	58.8 ± 4.2	57.3 ± 1.8	57.4 ± 1.2	57.5 ± 1.4	58.8 ± 1.5
MCH	17.9 ± 1.2	17.7 ± 0.3	18.3 ± 0.9	17.8 ± 0.7	17.9 ± 0.9
MCHC	30.5 ± 0.7	31.0 ± 1.2	31.9 ± 1.5	30.9 ± 1.0	30.3 ± 0.9
RDW	12.5 ± 1.0	12.0 ± 0.7	13.5 ± 2.2	11.7 ± 0.6	13.8 ± 1.9
WBC	9.6 ± 2.9	6.7 ± 2.6	7.1 ± 1.1	10.0 ± 2.2	10.8 ± 3.6
LYM%	74.5 ± 8.5	70.0 ± 8.7	66.2 ± 7.4	68.2 ± 6.6	68.7 ± 11.1
LYM#	7.3 ± 2.6	4.7 ± 1.8	4.2 ± 0.9	6.8 ± 1.3	7.1 ± 1.7
PLT	821 ± 164	827 ± 73	764 ± 136	813 ± 86	685 ± 180

	μM FANC/100 μL/25 g mice				
Female	0	0.6	2	6	20
RBC	8.3 ± 0.2	9.0 ± 0.7	8.6 ± 0.4	8.0 ± 0.6	8.7 ± 0.4
HGB	14.1 ± 0.5	14.1 ± 1.1	13.9 ± 0.4	13.2 ± 1.0	14.8 ± 0.5
HCT	45.1 ± 1.1	45.8 ± 2.8	45.1 ± 1.6	42.0 ± 2.9	46.8 ± 2.2
MCV	54.1 ± 1.1	51.1 ± 3.1	52.4 ± 1.2	52.2 ± 1.2	52.0 ± 1.7
MCH	17.1 ± 0.5	15.8 ± 1.3	16.1 ± 0.8	16.5 ± 0.9	16.4 ± 0.7
MCHC	31.4 ± 1.0	30.8 ± 0.9	30.7 ± 0.9	32.0 ± 1.0	31.6 ± 0.7
RDW	28.8 ± 0.5	29.1 ± 2.0	28.2 ± 0.5	28.0 ± 0.7	28.1 ± 0.8
WBC	7.6 ± 2.0	8.9 ± 4.3	5.7 ± 0.7	6.8 ± 1.7	10.9 ± 2.6
LYM%	73.4 ± 8.5	71.7 ± 14.9	72.8 ± 9.9	79.2 ± 8.4	72.7 ± 16.5
LYM#	5.2 ± 1.2	5.9 ± 1.4	4.0 ± 1.0	5.3 ± 1.2	6.6 ± 1.4
PLT	863 ± 171	1003 ± 165	951 ± 181	980 ± 159	888 ± 158

RBC: red blood cell (10^6/μL); HGB: hemoglobin (g/dL); HCT: hematocrit (%); MCV: mean corpuscular volume (fL); MCH: mean corpuscular hemoglobin (pg); MCHC: mean corpuscular hemoglobin concentration (g/dL); RDW: red blood cell distribution width (%); WBC: white blood cell (10^3/μL); LYM%: lymphocyte percentage (%); LYM#: lymphocyte count (10^3/μL); PLT: platelets (10^3/μL). Data are expressed as the mean ± SD (n=8).

Tables S6. Changes of hematological parameters of ICR mice feeding with FANC for 20 weeks

	μM FANC/100 μL/25 g mice				
Male	0	0.6	2	6	20
RBC	7.4 ± 1.5	7.7 ± 0.4	7.8 ± 0.6	7.9 ± 0.4	7.9 ± 0.4
HGB	13.2 ± 2.7	13.8 ± 0.7	13.7 ± 1.5	14.2 ± 0.5	14.5 ± 0.6
HCT	43.0 ± 7.1	43.9 ± 2.2	44.0 ± 4.4	45.4 ± 2.1	45.5 ± 2.0
MCV	59.2 ± 4.7	57.0 ± 1.8	56.4 ± 2.2	44.5 ± 38.2	57.4 ± 1.9
MCH	17.9 ± 0.8	17.9 ± 0.8	17.5 ± 0.9	14.1 ± 11.8	18.3 ± 0.8
MCHC	30.5 ± 2.7	31.4 ± 1.3	31.1 ± 0.5	31.3 ± 0.8	31.9 ± 1.0
RDW	13.9 ± 1.6	12.9 ± 1.6	13.2 ± 2.8	12.2 ± 0.8	13.6 ± 1.2
WBC	7.6 ± 2.1	8.0 ± 2.2	10.4 ± 4.9	9.8 ± 2.5	7.9 ± 2.3
LYM%	71.8 ± 8.2	68.8 ± 11.1	67.8 ± 13.1	64.9 ± 8.0	66.1 ± 13.7
LYM#	5.5 ± 1.9	5.4 ± 0.9	7.3 ± 4.7	6.4 ± 2.0	5.1 ± 1.3
PLT	806 ± 104	770 ± 126	752 ± 205	748 ± 207	707 ± 107

	μM FANC/100 μL/25 g mice				
Female	0	0.6	2	6	20
RBC	8.5 ± 0.5	8.6 ± 0.2	8.6 ± 0.6	8.7 ± 0.4	8.6 ± 0.4
HGB	14.3 ± 0.7	14.2 ± 0.4	14.4 ± 0.8	14.5 ± 1.1	14.4 ± 0.5
HCT	45.8 ± 2.5	45.6 ± 1.3	45.0 ± 3.0	45.8 ± 2.3	44.8 ± 1.7
MCV	53.9 ± 0.8	53.0 ± 1.0	52.9 ± 1.1	52.8 ± 1.6	52.3 ± 1.3
MCH	16.9 ± 0.6	16.5 ± 0.2	16.8 ± 0.7	16.7 ± 1.0	16.7 ± 0.7
MCHC	31.3 ± 1.0	31.1 ± 0.6	32.0 ± 1.1	31.5 ± 1.4	32.2 ± 0.8
RDW	27.2 ± 3.4	28.0 ± 0.4	28.4 ± 1.0	28.6 ± 0.6	28.9 ± 1.3
WBC	7.2 ± 2.8	9.2 ± 2.8	7.5 ± 2.1	8.2 ± 1.2	5.0 ± 0.9
LYM%	71.0 ± 10.3	74.1 ± 6.9	74.3 ± 7.5	76.3 ± 5.3	80.0 ± 7.1
LYM#	5.3 ± 2.5	6.7 ± 1.8	5.5 ± 1.2	6.2 ± 1.0	4.0 ± 1.0
PLT	728 ± 255	762 ± 306	923 ± 310	755 ± 140	887 ± 180

RBC: red blood cell (10^6/μL); HGB: hemoglobin (g/dL); HCT: hematocrit (%); MCV: mean corpuscular volume (fL); MCH: mean corpuscular hemoglobin (pg); MCHC: mean corpuscular hemoglobin concentration (g/dL); RDW: red blood cell distribution width (%); WBC: white blood cell (10^3/μL); LYM%: lymphocyte percentage (%); LYM#: lymphocyte count (10^3/μL); PLT: platelets (10^3/μL). Data are expressed as the mean ± SD (n=8).

Tables S7. Changes of hematological parameters of ICR mice feeding with FANC for 24 weeks

	μM FANC/100 μL/25 g mice				
Male	0	0.6	2	6	20
RBC	7.3 ± 1.8	7.3 ± 0.4	7.1 ± 0.8	6.9 ± 0.5	7.0 ± 0.9
HGB	13.0 ± 3.3	13.8 ± 0.8	13.3 ± 1.3	12.9 ± 0.9	13.1 ± 1.9
HCT	42.6 ± 8.2	40.6 ± 2.8	39.5 ± 4.7	38.5 ± 2.5	40.2 ± 5.9
MCV	60.1 ± 8.2	55.8 ± 1.5	55.3 ± 2.9	55.9 ± 1.2	56.9 ± 2.3
MCH	17.7 ± 0.3	18.6 ± 0.7	18.7 ± 1.3	18.4 ± 0.5	18.3 ± 0.8
MCHC	30.0 ± 3.2	33.8 ± 1.1	33.5 ± 1.5	33.4 ± 0.7	32.3 ± 0.6
RDW	14.1 ± 2.6	14.2 ± 0.9	18.0 ± 5.5	13.2 ± 1.1	14.5 ± 1.7
WBC	9.2 ± 2.7	9.5 ± 2.2	12.0 ± 10.9	11.6 ± 4.0	14.8 ± 11.8
LYM%	66.8 ± 12.8	76.2 ± 10.5	64.2 ± 13.7	69.5 ± 8.7	64.4 ± 11.0
LYM#	5.7 ± 1.6	7.1 ± 1.5	8.5 ± 10.4	8.0 ± 2.0	10.3 ± 11.3
PLT	600 ± 201	664 ± 152	472 ± 239	762 ± 81	410 ± 215

RBC: red blood cell (10^6/μL); HGB: hemoglobin (g/dL); HCT: hematocrit (%); MCV: mean corpuscular volume (fL); MCH: mean corpuscular hemoglobin (pg); MCHC: mean corpuscular hemoglobin concentration (g/dL); RDW: red blood cell distribution width (%); WBC: white blood cell (10^3/μL); LYM%: lymphocyte percentage (%); LYM#: lymphocyte count (10^3/μL); PLT: platelets (10^3/μL). Data are expressed as the mean ± SD (n=8).

Tables S8. Changes of hematological parameters of ICR mice feeding with FANC for 6 months and 4 weeks withdrawal period

	μM FANC/100 μL/25 g mice				
Male	0	0.6	2	6	20
RBC	6.1 ± 0.7	6.0 ± 0.1	4.8 ± 0.9	6.0 ± 0.4	6.1 ± 0.5
HGB	11.0 ± 1.0	10.6 ± 0.0	9.2 ± 1.5	9.2 ± 1.7	9.8 ± 1.5
HCT	25.0 ± 12.7	33.7 ± 0.5	28.8 ± 4.0	30.5 ± 5.0	31.2 ± 4.3
MCV	61.0 ± 12.7	55.8 ± 0.9	59.8 ± 2.9	50.5 ± 5.2	51.2 ± 3.3
MCH	17.9 ± 1.0	17.6 ± 0.4	19.0 ± 0.4	15.3 ± 1.9	16.1 ± 1.2
MCHC	30.1 ± 5.7	31.5 ± 0.4	31.8 ± 0.9	30.2 ± 0.7	31.5 ± 0.6
RDW	18.2 ± 5.2	13.3 ± 1.6	17.1 ± 2.6	17.9 ± 8.2	18.2 ± 5.1
WBC	4.9 ± 1.6	3.6 ± 0.6	5.6 ± 4.1	7.1 ± 6.6	6.8 ± 4.5
LYM%	64.2 ± 9.1	72.4 ± 10.9	72.6 ± 21.5	83.8 ± 12.2	77.1 ± 19.6
LYM#	2.9 ± 1.6	2.6 ± 0.6	4.6 ± 4.3	6.5 ± 6.9	5.9 ± 5.1
PLT	636 ± 207	745 ± 86	788 ± 262	743 ± 243	957 ± 263

	μM FANC/100 μL/25 g mice				
Female	0	0.6	2	6	20

RBC	6.3 ± 0.8	7.0 ± 0.1	7.0 ± 0.9	6.7 ± 1.1	7.5 ± 0.8
HGB	11.4 ± 1.3	11.4 ± 0.2	11.2 ± 0.8	10.7 ± 2.0	11.8 ± 0.7
HCT	32.4 ± 4.2	37.1 ± 0.6	36.2 ± 3.8	35.3 ± 6.7	39.0 ± 2.4
MCV	53.4 ± 0.6	52.7 ± 0.5	51.5 ± 2.4	52.5 ± 1.4	52.1 ± 2.4
MCH	16.5 ± 0.2	16.2 ± 0.3	15.9 ± 0.9	16.0 ± 0.5	15.8 ± 1.1
MCHC	30.9 ± 0.8	30.7 ± 0.3	30.9 ± 1.5	30.4 ± 0.9	30.4 ± 0.8
RDW	29.2 ± 1.2	27.9 ± 0.8	28.4 ± 0.7	27.8 ± 0.3	28.3 ± 1.5
WBC	3.5 ± 0.4	3.8 ± 1.1	2.3 ± 1.0	2.8 ± 1.2	3.8 ± 1.0
LYM%	86.7 ± 8.5	85.5 ± 4.7	75.0 ± 10.3	77.3 ± 13.9	78.4 ± 10.9
LYM#	3.0 ± 0.3	3.3 ± 1.1	1.8 ± 1.1	2.1 ± 0.5	3.0 ± 0.8
PLT	882 ± 117	958 ± 93	1029 ± 147	943 ± 378	988 ± 128

RBC: red blood cell (10^6/μL); HGB: hemoglobin (g/dL); HCT: hematocrit (%); MCV: mean corpuscular volume (fL); MCH: mean corpuscular hemoglobin (pg); MCHC: mean corpuscular hemoglobin concentration (g/dL); RDW: red blood cell distribution width (%); WBC: white blood cell (10^3/μL); LYM%: lymphocyte percentage (%); LYM#: lymphocyte count (10^3/μL); PLT: platelets (10^3/μL). Data are expressed as the mean ± SD (n=3).
